# Needs and Requirements in the Designing of Mobile Interventions for Patients With Peripheral Arterial Disease: Questionnaire Study

**DOI:** 10.2196/15669

**Published:** 2020-08-04

**Authors:** Julia Lortz, Jan Simanovski, Tabea Kuether, Ilonka Kreitschmann-Andermahr, Greta Ullrich, Martin Steinmetz, Christos Rammos, Rolf Alexander Jánosi, Susanne Moebus, Tienush Rassaf, Katrin Paldán

**Affiliations:** 1 Department of Cardiology and Vascular Medicine West-German Heart and Vascular Center Essen University of Duisburg-Essen Essen Germany; 2 Centre for Urban Epidemiology Institute for Medical Informatics, Biometry and Epidemiology University of Duisburg-Essen Essen Germany; 3 Centre of Competence Personal Analytics at the University of Duisburg-Essen Department of Engineering Sciences University of Duisburg-Essen Duisburg Germany; 4 Department of Neurosurgery University of Duisburg-Essen Essen Germany

**Keywords:** peripheral arterial disease, mHealth, digital intervention, supervised exercise therapy, smartphone usage

## Abstract

**Background:**

The development of mobile interventions for noncommunicable diseases has increased in recent years. However, there is a dearth of apps for patients with peripheral arterial disease (PAD), who frequently have an impaired ability to walk.

**Objective:**

Using a patient-centered approach for the development of mobile interventions, we aim to describe the needs and requirements of patients with PAD regarding the overall care situation and the use of mobile interventions to perform supervised exercise therapy (SET).

**Methods:**

A questionnaire survey was conducted in addition to a clinical examination at the vascular outpatient clinic of the West-German Heart and Vascular Center of the University Clinic Essen in Germany. Patients with diagnosed PAD were asked to answer questions on sociodemographic characteristics, PAD-related need for support, satisfaction with their health care situation, smartphone and app use, and requirements for the design of mobile interventions to support SET.

**Results:**

Overall, a need for better support of patients with diagnosed PAD was identified. In total, 59.2% (n=180) expressed their desire for more support for their disease. Patients (n=304) had a mean age of 67 years and half of them (n=157, 51.6%) were smartphone users. We noted an interest in smartphone-supported SET, even for people who did not currently use a smartphone. “Information,” “feedback,” “choosing goals,” and “interaction with physicians and therapists” were rated the most relevant components of a potential app.

**Conclusions:**

A need for the support of patients with PAD was determined. This was particularly evident with regard to disease literacy and the performance of SET. Based on a detailed description of patient characteristics, proposals for the design of mobile interventions adapted to the needs and requirements of patients can be derived.

## Introduction

Circulatory disorders of peripheral arteries due to atherosclerotic lesions are the third most frequent manifestation of atherosclerotic disease after its manifestation in coronary and cerebrovascular arteries [1]. Nevertheless, peripheral artery disease (PAD) causes the highest treatment costs for health care providers of all cardiovascular disorders [2]. The prevalence of PAD increases with age and affects a substantial proportion of the elderly population (>20% in those aged >80 years) [1,3]. Additionally, PAD is linked to higher morbidity and mortality and leads to a significantly reduced quality of life including daily life restrictions [4], ranging from mild impairment in walking distance to limb amputations [5,6].

One recommendation of the current guidelines is supervised exercise therapy (SET) or a supervised exercise program (SEP) [3,7]. For better readability, we refer to both SET and SEP as SET from here on. Regularly reaching the pain threshold leads to better leg perfusion and makes SET one of the most effective (both medically and economically) conservative therapies for extending pain-free walking distance [7-10]. The regular performance of SET has already been proven to be associated with decreased mortality and also results in an improvement in functional health and quality of life [3,10-12].

Recent studies have shown that patient empowerment helps to increase therapy adherence. This is mainly achieved through gaining greater control in health decisions [13-15]. The needs and preferences of the patient have to receive more attention and patients should be involved more closely in the process of care. A deeper exploration of clinical and demographic characteristics may influence the response to SET, help to overcome barriers and allow for the possibility of designing tailor-made solutions to implement SET in a patient’s everyday life [10].

Mobile health (mHealth) technologies provide digital solutions to close gaps in care [16,17]. The use of mobile devices (eg, smartwatches and smartphones) permits the monitoring of health data that far exceeds the information gathered in a brief clinical encounter [18]. Based on persuasive design aspects, mobile devices also offer opportunities to support patients’ health-related behavior [19]. The development of mHealth interventions for noncommunicable diseases has progressively received attention in recent years. However, patients with PAD and their specific requirements (due to a frequently impaired ability to walk) have been neglected thus far. Current gaps in research arise from the fact that no specific apps are designed for patients with PAD. Studies either focus on the general aspects of cardiovascular health including (remote) counselling [20], or use nonspecific mHealth technologies that aim to raise the level of general activity [21], instead of providing a PAD-specific approach in promoting SET. PAD-specific mHealth solutions are not currently available, but their development should be informed by first identifying the requirements of the PAD-population.

As a first step in a patient-centered approach to develop PAD-specific mobile interventions, we describe the needs and requirements from a patient perspective.

The aim of the study was to determine the needs and requirements of patients with PAD. This included their overall care situation and the potential use of mobile interventions.

In addition to the clinical examination, we answer the following research questions:

1. What is the current perception of medical care in patients with PAD? Can a need for medical support be determined among the study participants?
2. Do patients with PAD currently use smartphones and apps? What are the characteristics of smartphone users and nonusers?
3. What are the requirements for the design of mobile interventions to support patients with PAD in performing supervised exercise therapy?

## Methods

### Study Design and Patient Recruitment

In addition to the clinical examination, we conducted a questionnaire-based survey at the vascular outpatient clinic of the West-German Heart and Vascular Center Essen of the University Clinic Essen, Germany. This clinic treats more than 1500 patients with PAD annually. Patients were recruited between September and December 2018.

### Inclusion and Exclusion Criteria

Consecutively, patients with diagnosed PAD were asked to participate in this study. The inclusion criteria were male or female patients aged 18 or older with PAD. PAD had to be diagnosed at least 3 months prior to the study.

Furthermore, patients were excluded if they were unable to complete the questionnaire themselves (eg, severe dementia or cognitive dysfunction). We also excluded individuals who did not have sufficient knowledge of the German language.

### Sample Size and Basic Sociodemographic Characteristics

In total, we surveyed 304 patients with PAD. Two-thirds of the patients were men (n=203, 66.8%; Table 1). The participants were aged between 41 and 90 years (mean 67 years, SD 10.21). In total, 133 (46.5%) of the participants had an upper-medium educational attainment (12 to 13 years of education), and 62 (21.7%) had a lower educational attainment (≤12 years of education). Overall, 24 (7.9%) said they did not have a secondary school graduation certificate.

**Table 1:** Sociodemographic characteristics of patients with peripheral arterial disease (PAD) divided into all patients, smartphone users, and non–smartphone users.

Sociodemographic characteristics		All patients, n (%)		Smartphone users, n (%)		Non–smartphone users, n (%)		*P* value (*χ*^2^ value)	
**Sex**		n=304		n=157		n=147			
	Male		203 (66.8)^a^		106 (52.2)^b^		97 (47.8)^b^		.73 (0.121)
**Age (years)**		n=301		n=155		n=146			
	40-49		18 (6.0)		11 (61.1)		7 (38.9)		.74 (0.113)
	50-59		58 (19.3)		34 (58.6)		24 (41.4)		.46 (0.556)
	60-69		102 (33.9)		64 (62.7)		38 (37.3)		.09 (2.870)
	70-79		85 (28.2)		35 (41.2)		50 (58.8)		.32 (1.002)
	≥80		37 (12.3)		11 (29.7)		26 (70.3)		.12 (2.382)
**Educational attainment (years)**		n=286		n=151		n=135			
	<10		40 (14.0)		19 (12.6)		21 (15.6)		.91 (0.012)
	10**-**11		22 (7.7)		13 (8.6)		9 (6.7)		.76 (0.091)
	12**-**13		133 (46.5)		67 (44.4)		66 (48.9)		.09 (0.001)
	14**-**17		66 (23.1)		35 (23.2)		31 (23.0)		.32 (0.030)
	>17		25 (8.7)		17 (11.3)		8 (5.9)		.12 (1.013)
**Employment status**		n=304		n=157		n=147			
	Currently employed		77 (25.3)		43 (55.8)		34 (44.2)		.57 (0.319)
	Retired		140 (46.1)		86 (42.9)		94 (57.1)		.75 (0.100)
	Retired due to illness		40 (13.2)		26 (65.0)		14 (35.0)		.26 (0.258)
**Burden of PAD**		n=304		n=157		n=147			
	Not at all		21 (6.9)		7 (33.3)		14 (66.7)		.43 (0.612)
	A little		49 (16.1)		23 (46.9)		26 (53.1)		.92 (0.102)
	Average		74 (24.3)		36 (48.6)		38 (51.4)		.97 (0.002)
	Fair		98 (32.2)		57 (58.2)		41 (41.8)		.32 (1.007)
	Great		62 (20.4)		34 (54.8)		28 (45.2)		.72 (0.129)
Burden of disease		1.36 (0.76)		1.45 (0.73)		1.25 (0.76)		.02 (2.33)	
Burden of environmental conditions		0.90 (0.63)		1.02 (0.58)		0.77 (0.64)		<.001 (3.377)	

^a^The percentage is based on the number of all responses for the associated sociodemographic characteristic (sex, age, educational attainment, employment status, and burden of PAD).

^b^The percentage is based on the total number of observations within the associated sociodemographic characteristic group.

### Ethics

The study was conducted in accordance with the Declaration of Helsinki, and the protocol was approved by the local ethics committee at the Faculty of Medicine of the University Duisburg-Essen. Patient records were deidentified and analyzed anonymously. Written consent was obtained from each patient included in the study.

### Measurements: Questionnaire

The questionnaire was prepared specifically for this study and was pretested on 5 PAD patients not included in the study sample. The pretest did not reveal the need for any changes.

The 10-page questionnaire encompasses a total of 31 questions on sociodemographic characteristics; subjective burden of disease and PAD-related care situations; subjective burden of environmental conditions; implementation and feasibility of SET; mobile or app usage; interests and knowledge regarding SET and medication; and the need for support and satisfaction with the health care situation. In the questionnaire, we used the term supervised walking training instead of the technical term supervised exercise therapy (SET) because the German word “Gehtraining” is more established in clinical practice.

The questionnaire included dichotomous and 5-point assessments similar to the Likert scale, adapted response scales, and open-ended questions. The questionnaire (English translation) is provided in Multimedia Appendix 1.

The thematic structure of the questionnaire included the following topics:

- Need for support
- Satisfaction with health care situation
- Sociodemographic characteristics
- Burden of environmental conditions
- Burden of PAD and other diseases
- Pain-free walking distance
- Clinical characteristics
- Preferences regarding offers to support patients with PAD
- Smartphone usage, knowledge about health apps, and health app usage
- Design categories in health apps to support patients with PAD

The detailed classification of the topics is shown in Multimedia Appendix 2. The items were chosen to measure the current level of burden in terms of PAD and other diseases. Relevant characteristics to classify PAD severity were also interrogated. In addition, it should be examined to what extent digital interventions represent a possible approach to support affected patients. Sociodemographic characteristics should provide information about the special requirements of different subgroups.

### Analysis

We performed descriptive data analysis using SPSS (Version 23; IBM Corp). Variables are presented as frequencies and percentages or as means and standard deviations. Variables were compared using an unpaired *t* test or chi-square test, and a one-way analysis of variance when more than two groups were compared. Values of *P*<.05 were considered statistically significant. For comparative analyses of normally distributed variables, parametric tests such as the Student *t* test were used to test the assumption of homogeneity or nonhomogeneity of variance.

## Results

### Research Question 1: What Perceptions Do Patients With PAD Have of Their Medical Care? Do Study Participants Indicate a Need for Medical Support?

Overall, the need for more medical support in patients with PAD was identified (Table 2). Two-thirds (n=180, 59.2%) of the surveyed patients expressed their desire for more support in regard to their disease. More than half (n=162, 53.2%) of the patients were not very satisfied with their health care situation. The question regarding patient knowledge about current medical therapies and recommendations regarding SET indicated both a poor level of patient care and information. Two-thirds (n=198, 65.1%) of the participants stated that they did not know if they were taking medication to treat their PAD. The lack of suitable medication occurred in all Fontaine stages (I: n=101, 33.3%; IIa: n=34, 11.1%; IIb: n=135, 44.4%; and IV: n=34, 11.1%), although medication is recommended for all stages. The vast majority of patients (n=264, 86.8%) reported that their physician did not explain why their prescribed medication was important. Two-thirds of the patients (n=194, 63.8%) answered “no” to the question of whether they had already been recommended to perform walking training for the treatment of PAD. More than half of the patients (n=163, 53.6%) were even not familiar with the term “supervised exercise therapy.” Only 26% (n=79) said they already performed walking training on a regular basis.

**Table 2:** Current perceptions of central aspects of medical care and need for support in patients with peripheral arterial disease.

Items		Total responses, n (%)	40-49 years, n (%)	50-59 years, n (%)	60-69 years, n (%)	70-79 years, n (%)	>80 years, n (%)
		N=304	n=18	n=58	n=102	n=85	n=38
**Overall need for support**							
	Yes	180 (59.2)^a^	8 (44.5)^b^	31 (53.4)^b^	60 (58.8)^b^	49 (57.6)^b^	29 (76.3)
**Health care satisfaction**							
	Completely dissatisfied	50 (16.4)	2 (11.1)	16 (27.6)	12 (11.8)	10 (11.8)	9 (23.7)
	Rather dissatisfied	112 (36.8)	8 (44.4)	20 (34.5)	37 (36.3)	36 (42.4)	11 (28.9)
	Neither dissatisfied nor satisfied	63 (20.7)	2 (11.1)	12 (20.7)	24 (23.5)	20 (23.5)	5 (13.2)
	Rather satisfied	46 (15.1)	2 (11.1)	7 (12.1)	18 (17.6)	12 (14.1)	6 (15.8)
	Very satisfied	33 (10.9)	4 (22.2)	3 (5.2)	11 (10.8)	7 (8.2)	7 (18.4)
**Medication for peripheral arterial disease**							
	Yes	77 (25.3)	8 (44.4)	14 (24.1)	24 (23.5)	22 (25.9)	8 (21.1)
**Information about medication**							
	Yes	40 (13.2)	3 (16.7)	8 (13.8)	9 (8.8)	14 (16.5)	6 (15.8)
**Recommendation for supervised walking training^c^**							
	Yes	13 (4.3)	0 (0)	3 (5.2)	4 (3.9%	3 (3.5)	3 (7.9)
**Information about supervised walking training^c^**							
	Yes	36 (11.8)	2 (11.1)	5 (8.6)	15 (14.7)	9 (10.6)	5 (13.2)
**Performance of supervised walking training^c^**							
	Yes	79 (26.0)	3 (16.7)	16 (27.6)	32 (31.4)	21 (24.7)	7 (18.4)

^a^The percentage is based on the total number of responses for the associated item.

^b^The percentage is based on the total number of people in the age group.

^c^In the questionnaire, we used the term supervised walking training instead of the technical term supervised exercise therapy (SET) because the German word “Gehtraining” is more established in clinical practice.

### Research Question 2: Do Patients with PAD Currently Use Smartphones and Apps? What are the Characteristics of Smartphone Users and Non–Smartphone Users?

Table 1 presents the following sociodemographic characteristics: (1) the participants who use a smartphone, (2) the participants who do not use a smartphone, and (3) a summary of the entire study sample. In total, 304 patients provided information on whether they use a smartphone.

Half of the patients (n=157, 51.6%) were smartphone users. Health apps were used by only a minority of patients (n=17, 5.7%). However, almost half (n=146, 48%) of all participants said they had already heard about health apps for smartphones that are designed to support health improvement.

The proportion of men and women who used a smartphone was comparable (n=159, 52.2% versus n=154, 50.5%, *P*=.73, *χ^2^*=0.728). Patients aged between 40 and 69 years were more likely to use a smartphone than not (n=21, 61.2% were active users). This trend changed in patients aged 70 years or older. Two-thirds of the patients aged 70 years or older did not use a smartphone (n=116, 38.1% were active users, *P*=.07, *χ^2^*=3.262).

Among those who had a low to upper-medium educational attainment (≤17 years of education), we did not see notable differences between users of smartphones and nonusers (*P*=.83, *χ^2^*=0.048). However, in patients with a high educational attainment (>17 years; equivalent to a university degree), we found a tendency toward higher smartphone use, but this was not statistically significant (n=4, 11.3% versus n=18, 5.9%, *P*=.31, *χ^2^*=1.013). Three-fourths of the participants (n=234, 76.9%) were not currently employed; of these, 94 (63.9%) were retired. The most frequent reason for retirement was having reached retirement age (n=165, 54.4%). Only 14 patients (9.5%) had retired due to illness.

Overall, patients tended to feel “quite burdened” (n=98, 32.2%) to “very burdened” (n=62, 20.4%) by their PAD. In addition to PAD, patients were mainly affected by diseases of the musculoskeletal system (mean 2.32, SD 1.63), diseases of the cardiovascular system (mean 2.14, SD 1.50) and respiratory diseases (mean 1.54, SD 1.43).

The burden of environmental conditions was indicated by a mean of 0.90 (SD 0.63), which corresponds to a low burden of environmental conditions. Patients were mainly affected by environmental conditions such as “financial worries” (mean 1.32, SD 1.28), followed by “constant responsibility for their family” (mean 1.23, SD 1.22) and “household” (mean 1.23, SD 5.85), but the standard deviation for the “household” item was conspicuously large. The group of patients without a smartphone felt somewhat less burdened, both in terms of burden of disease and burden of environmental conditions (*P*=.02, *t*=2.330 and *P*<.001, *t*=3.377, respectively).

In Table 3, we report the differences in health status and risk factors between participants with and without smartphone use. The pain-free walking distance is a relevant marker for compensated PAD. One-third (n=101, 33.2%) of participants said that they could walk <200 meters without pain. Additionally, 28% (n=85) of the participants reported they could walk between 200 and 1000 meters, and 29% (n=88) were hardly restricted and reported a pain-free walking distance of more than 1000 meters (n=30 or 9.9% chose the answer option “I do not know”). The two groups (smartphone users and nonusers) were comparable with regard to their pain-free walking distance and did not differ substantially.

**Table 3:** Health status and risk factors of patients with peripheral arterial disease divided into all patients, smartphone users, and non–smartphone users.

Health status or risk factor		All patients	Smartphone users	Non–smartphone users	*P* value (*χ*^*2*^ value)
**Pain-free walking distance, mean (SD)**		n=304	n=157	n=147	
	<200 m	101 (33.2)^a^	53 (52.5)^b^	48 (47.5)^b^	.83 (0.045)
	200-1000 m	85 (28.0)	47 (55.3)	38 (44.7)	.59 (0.289)
	>1000 m	88 (28.9)	43 (48.9)	45 (51.1)	.97 (0.001)
	I do not know	30 (9.9)	14 (46.7)	16 (53.3)	.95 (0.004)
**Disease severity according to Fontaine [22], mean (SD)**		n=299	n=155	n=144	
	Stage I	125 (41.8)	66 (52.8)	59 (47.2)	.75 (0.100)
	Stage IIa	44 (14.7)	28 (63.6)	16 (36.4)	.28 (1.158)
	Stage IIb	103 (34.4)	52 (50.5)	51 (49.5)	.97 (0.001)
	Stage III	7 (2.3)	5 (71.4)	2 (28.6)	.78 (0.075)
	Stage IV	20 (6.7)	4 (20.0)	16 (80.0)	.10 (2.747)
**BMI, mean (SD)**		n=176	n=90	n=86	
	Underweight (**<**18.5 kg/m^2^)	7 (4.0)	2 (2.2)	5 (5.8)	.78 (0.075)
	Normal (18.5**-**24.9 kg/m^2^)	55 (31.2)	27 (30.0)	28 (32.6)	.96 (0.002)
	Overweight (25.0**-**29.9 kg/m^2^)	69 (39.2)	36 (52.2)	33 (38.4)	.93 (0.007)
	Obese (**>**30 kg/m^2^)	45 (25.6)	25 (27.8)	20 (23.3)	.75 (0.100)
**Currently smoking, mean (SD)**		n=275	n=142	n=133	
	Yes	86 (31.3)	47 (33.1)	39 (29.3)	.65 (0.210)
	Not anymore	39 (14.2)	19 (13.4)	20 (15.0)	.96 (0.003)

^a^Percentage is based on the number of responses for the associated health status or risk factor (pain-free walking distance, disease severity, BMI, smoking).

^b^Percentage is based on the total number of observations within the associated group of health outcomes or risk factors, regardless of smartphone use.

Based on the severity of the disease, 42% (n=128) were in Fontaine Stage I (corresponding to mild PAD), 15% (n=46) were in Stage IIa, 34% (n=103) were in Stage IIb, 2% (n=6) were in Stage III, and 7% (n=21) were in Stage IV (corresponding to very severe PAD). On average, patients in the smartphone group (mean 2.05, SD 1.06) and patients in the non–smartphone group (mean 2.30, SD 1.31) had mild PAD. However, in the non–smartphone group, there were more cases of severe PAD (Stage IV, 20% [n=61] versus 80% [n=243], *P*=.10).

In total, more than one-third (n=119, 39.2%) of the participants were overweight, and an additional 26% (n=79) were obese. Normal weight was documented in 31% (n=94) of the participants, and 4% (n=12) of the participants were underweight.

Almost half of the participants (n=140, 46%) had smoked at one stage of their life, and of these participants, 31% (n=94) were current smokers and 14% (n=43) had already quit smoking. With regard to smoking behavior, the smartphone users and nonusers did not show substantial differences (*P*=.65 and *P*=.96, respectively).

### Research Question 3: What are the Requirements for the Design of Mobile Interventions to Support Patients with PAD?

**Figure 1:**
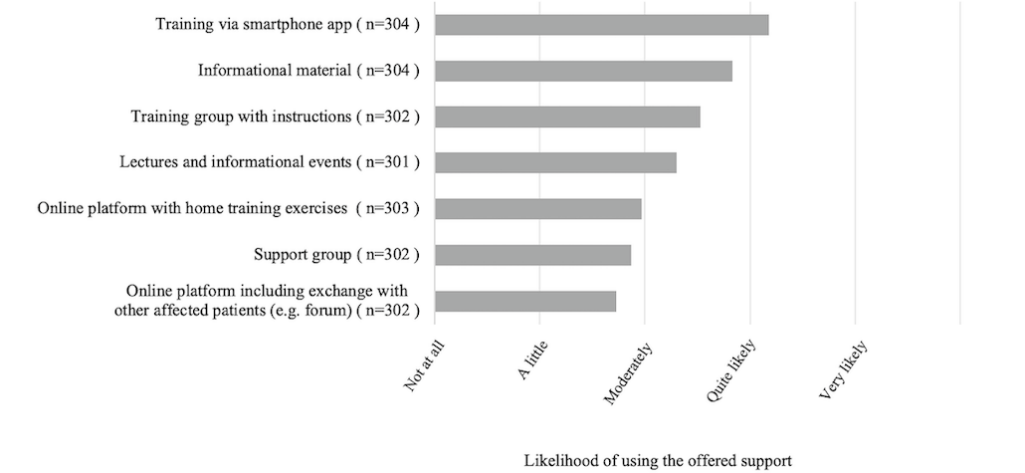
Descriptive analysis of user preferences in terms of offered app components for patients with peripheral arterial disease (refers to Question 15 of the questionnaire). Note that multiple choices were possible.

When asked how likely it was that they would use the listed services, participants indicated that they were most likely to use a “training app” on their smartphone (mean 3.18, SD 1.28), followed by “informational material” (mean 2.83, SD 1.48) and “training groups with instructions” (mean 2.53, SD 1.45). “Online platforms” (mean 1.73, SD 1.10) and “support groups” (mean 1.87, SD 1.87) were the response options that participants indicated they were least likely to use. The probability of making use of the listed options for patients with PAD is summarized in Figure 1.

**Figure 2:**
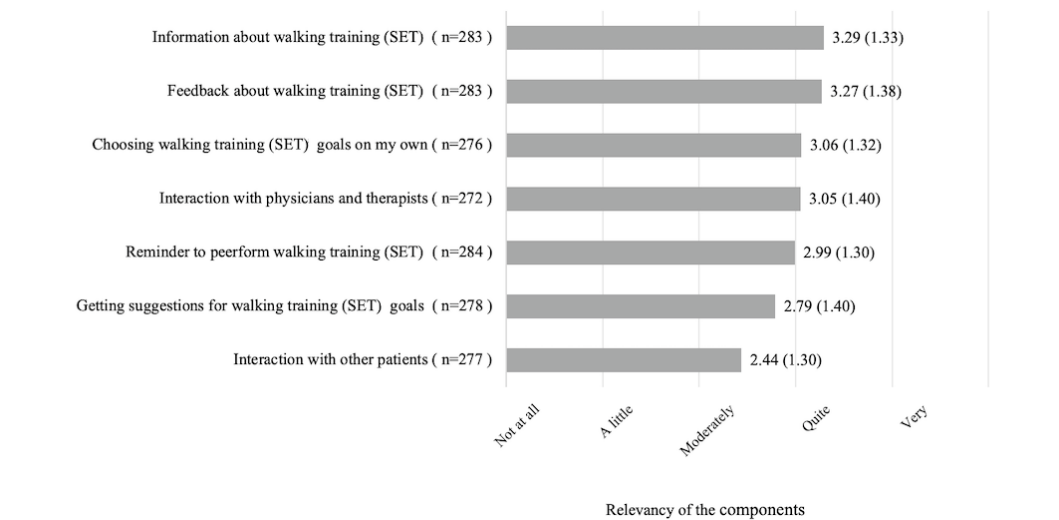
Descriptive analysis of user-reported relevance in terms of health app components that would assist patients with peripheral arterial disease performing supervised walking training. The analysis refers to question 15 of the questionnaire. Note that multiple choices were possible. In the questionnaire, we used the term "supervised walking training" instead of the technical term "supervised exercise therapy" (SET) because the German word “Gehtraining” is more established in clinical practice.

Figure 2 shows the ranking of the components of health apps that can be used to support patients. The most relevant components were “information” (mean 3.29, SD 1.33), “feedback” (mean 3.27, SD 1.38), “choosing goals” (mean 3.06, SD 1.32) and “interaction with physicians and therapists” (mean 3.05, SD 1.40). The least relevant component was “interaction with other patients” (mean 2.44, SD 1.30).

Table 4 shows the ranking of components of health apps that can be used to support patients depending on disease severity according to Fontaine stage [22]. The importance of the components were most likely to be seen in participants with Stage IIa across all components of health apps that support patients with PAD, although the averages did not surpass “moderately” to “fairly important.” All components were rated highest and at least moderately important by patients with Stage IIa. Although patients with Stage I were, by definition, not restricted in everyday life, support components such as “information” (mean 3.32, SD 1.30), “feedback” (mean 3.36, SD 1.36), “choosing goals” (mean 3.14, SD 1.36), and “interaction with physicians and therapists” (mean 3.10, SD 1.42) were seen to be moderately to fairly important for patients in Stage I with regard to performing SET using a health app. The performed variance analysis showed a significant difference between Fontaine stages for “interaction with physicians and therapists” (*P*=.04, *F*=4.231) and “getting suggestions for walking training (SET) goals” (*P*=.03, *F*=5.026). There was a trend toward differences between the Fontaine stages for “reminder to perform walking training (SET)” and “choosing walking training (SET) goals on my own” but these differences were not statistically significant (*P*=.06, *F*=3.668 and *P*=.08, *F*=3.034, respectively).

Age was only found to have an effect on the answer to “reminder to perform walking training (SET).” The older the participants were, the more they preferred a reminder function of a health app (*P*=.02, *F*=5.933). Other effects of age were not found in terms of supporting app components.

The ranking of the individual components differed slightly depending on the severity of the disease. For participants in Stage IIa, the ranking was as follows: (1) interaction with physicians and therapists, (2) information about SET, (3) feedback about SET, (4) choosing goals, (5) suggestions for goal setting, (6) reminders, and (7) interaction with other patients. Interaction with physicians and therapists was less important for patients in Stages I, IIb, and IV.

Information regarding SET and feedback about SET were moderately to fairly important for patients regardless of disease stage. Interactions with other patients were considered least important by participants in Stages I to III. For Stage IV participants, interactions with other participants were considered more important than choosing goals, reminders, and suggestions for goal setting. The core results of the study are summarized in Table 5.

**Table 4:** Relevance of components of health apps to support patients with peripheral arterial disease by disease severity according to Fontaine stages [22].

Components of health apps to support patients	Stage I		Stage IIa		Stage IIb		Stage III		Stage IV	
	Patients, n	Mean (SD)	Patients, n	Mean (SD)	Patients, n	Mean (SD)	Patients, n	Mean (SD)	Patients, n	Mean (SD)
Information about walking training^a^	113	3.32 (1.30)	42	3.66 (1.26)	97	3.28 (1.37)	6	2.66 (1.21)	20	2.80 (1.15)
Feedback about walking training	111	3.36 (1.36)	42	3.59 (1.30)	99	3.12 (1.40)	7	2.57 (1.27)	19	3.36 (1.38)
Choosing walking training goals on my own	110	3.14 (1.36)	40	3.35 (1.16)	95	3.03 (1.30)	6	2.16 (1.47)	20	2.70 (1.26)
Interaction with physicians and therapists	107	3.10 (1.42)	39	3.71 (1.21)	95	2.91 (1.38)	7	2.42 (1.27)	20	2.75 (1.25)
Reminder to perform walking training	112	3.08 (1.32)	41	3.17 (1.30)	100	2.99 (1.26)	7	2.57 (1.27)	19	2.42 (1.12)
Getting suggestions for walking training goals	110	2.90 (1.41)	40	3.32 (1.36)	98	2.69 (1.40)	7	2.00 (1.15)	19	2.31 (1.00)
Interaction with other patients	111	2.39 (1.28)	39	3.00 (1.31)	97	2.30 (1.27)	7	1.71 (1.11)	19	2.73 (1.19)

^a^In the questionnaire, we used the term “supervised walking training” instead of the technical term “supervised exercise therapy” because the German word “Gehtraining” is more established in clinical practice.

**Table 5:** Summary of core results.

Research question	Summary of core results
1	A need for support was determined. Receiving more educational health information, increased support in the form of prescribed medication, and help in terms of implementing supervised exercise therapy (SET) are the most desired actions for improving the care of patients with PAD.
2	Half of the participants use smartphones. For them, mobile interventions to support SET and medication can be a relevant treatment component. Patients aged >70 years are less likely to use smartphones than younger patients. With regard to characteristics such as sex, education, profession, BMI, smoking behavior, exposure to illness or the environment, or the current state of illness, the data did not reveal any significant differences between smartphone users and nonusers within the patient population.
3	Interest in smartphone-supported training is present, even for people who do not currently use a smartphone. Health app components such as “information,” “monitoring,” and “feedback” were the most relevant for patients with PAD. Other components such as “choosing goals,” “interaction with physicians and therapists,” “interaction with other patients,” and “reminders and suggestions for goal setting” were less relevant for the patients and should be selectable on demand according to patient preference.

## Discussion

### There Is a Need for Supporting the Care of Patients With PAD

More than half (53.2%) of the participants were less than satisfied or completely unsatisfied with their health care situation. Patients do not feel well-informed enough in terms of SET and their prescribed medication. Since both are cornerstones in the treatment of PAD, this finding is alarming in terms of secondary prevention and long-term outcomes. The lack of educational background is expected to be associated with poor medication and exercise compliance, impeding the successful empowerment of patients. Previous research found that mHealth interventions improve adherence to prescribed medication in patients with cardiovascular disease [23].

Our results show an evident need for action to support patients with PAD in secondary prevention. A major goal should include patient empowerment. The demand for more support was found in all subgroups, independent of age or severity of disease. Institutional barriers in particular (eg, a lack of training groups and primary health care providers providing care to patients with PAD) limit the likelihood of an adequate health care offer for affected patients. Previous studies already reported the undersupply of primary health care for patients with PAD in general as well as those from various sociodemographic backgrounds [24,25].

### A Call for Patient-Centered Mobile Interventions

Personal barriers are primarily linked to poor knowledge about the disease and low empowerment. Mobile interventions might play an ever-increasing role, since they are widely accessible and have a low threshold for access. Time resources for consultations between patients and doctors are limited. In clinical practice, lifestyle recommendations are made within a few minutes. To increase the probability of patients’ adherence and their empowerment to take responsibility for their own health, personalized approaches are promising [26]. However, these require the involvement of the patient. The analysis of patient characteristics, smartphone use, and requirements for support measures is a first step to identify patient needs. On the basis of surveys focusing on patients’ specific needs and requirements, patient-centered interventions can be developed; at the same time, deficiencies in the current health care situation can be identified and potentially improved.

The use of patient-centered methods to develop persuasive strategies for mHealth interventions [27], as well as the conception and implementation of analog interventions, can help to take different social groups into account, with respect to their specific and individual needs.

### Requirements for the Design of Mobile Interventions

The idea of using a training app was of strong interest, even to participants who currently do not use smartphones. Based on this preference for digital support, the need to design and implement motivating tools that provide educational information was identified. In this setting, the analysis of assessed data regarding usage and user preferences might also be helpful in the feedback process. The current study also demonstrated patients’ priorities regarding important features, such as the opportunity to set individual goals or to get in touch with professionals, including physicians or therapists. Conspicuously, the offered support of interactions with other patients tended to perform poorly, both as a proposed digital chat component in an app and as a component of an analog intervention in the sense of a support group. A previous study showed a high acceptance of electronic health information and disease-related community forums in patients with PAD [28]. This discrepancy might result from the sole query regarding the preferences of interaction between patients without further explanation. On the other hand, one might deduce that tools do not primarily have to offer (specific) messenger services to enable and support networking between the patients. However, the opportunity for patients to compare and compete with each other (within an app) was not reviewed. Further research is needed to evaluate this kind of digital support.

### Modular Concepts Adapted to the Needs of Patients Appear Promising

Depending on the severity of their disease, the participants’ ranking of useful components within a digital intervention app differed slightly. Although “information,” “monitoring,” and “feedback” should be fixed components within apps that support patients with PAD, other components, such as “goal selection,” “interaction with physicians or therapists,” “interaction with other patients,” “reminders for structured walking training,” and “suggestions for individual goals” can be offered additionally, as a voluntary, selectable feature according to patients’ preferences.

In addition to tools for the implementation of SET, supplementary components that support medication use, healthier nutrition, or cessation of smoking appear useful. Considering the BMI of our study population compared to the German population in 2017 (overweight: 35.9%; obese: 18.1%), the sample was above the national average [29]. Therefore, a combination of components that promote more exercise and a healthier lifestyle including nutrition may contribute to weight management. Weight reduction also reduces the risk of multimorbidity, which was a common burden in our cohort.

### Challenges to Face

The efficacy of digital interventions is significantly influenced by an individual’s engagement with, for example, a specific app [30]. In particular, mHealth technologies face one major limiting factor in terms of long-term engagement: the vast majority do not exceed 6 months of regular app use [31,32]. This phenomenon does not occur only in healthy subjects who aim for a healthier lifestyle, but also in secondary prevention, where long-term behavioral changes toward a more active lifestyle are associated with health benefits [33,34]. Strategies that improve user engagement linked to these technologies may include elements of gamification [35] and devices deeply intertwined with everyday life, such as smartphones or wearables [36], that deliver instant feedback of good behavior. Our results showed a Fontaine stage–dependent decline in interest in interactions with physicians and therapists, and suggested walking training (SET) goals. This effect might be linked to a higher frustration level in patients with an advanced disease stage [37] and emphasizes the importance of early diagnosis and treatment.

Another consideration is the age of the target group, which often includes older patients with noncommunicable diseases. The mean age of participants in this study was 67 years. More than half of the patients aged 70 years or older were not reachable by mobile interventions. This finding has to be taken into consideration when designing digital interventions. Similar results regarding the use of digital interventions in older patients were previously observed [38]. Nevertheless, older patients are not to be neglected in terms of the development of digital interventions as the aging population is expected to become increasingly accustomed to the use of smart technologies [39]. Further physical impairments like diminished eyesight related to diabetes or deteriorated motor skills have to be taken into account, since they occur more often with increasing age. Motivational and cognitive barriers of older adults are major challenges [40,41] and need to be investigated more in detail. In this study, we found that a reminder to perform walking training (SET) would be of special interest in the older population, but other differences between the younger and older patients were not seen. Since this study did not focus on age-related preferences of app development in detail, further research is needed to investigate special needs and requirements in older adults.

### Limitations and Future Work

This analysis merely serves as an empirically sound description of the addressed problem and identifies approaches to improve the care of patients with PAD. This study included only a small sample; thus, the results cannot be generalized.

The study focused on a selection of personal characteristics to avoid time-consuming interviews before starting the actual clinical examination. Other characteristics of patients with PAD that may affect the need for and responsiveness to interventions supporting SET in daily living (eg, self-efficacy, motivation to change, race/ethnicity, income, social capital) were not examined. Based on the present study findings, we developed an app to support SET for patients with PAD [42], but the results are pending.

This study also did not address environmental factors. This is a potential point of criticism. With regard to health, in addition to personal characteristics, environmental characteristics play an important role in the implementation of health-promoting and therapy-compliant behaviors [43]. To obtain health-related environmental data (eg, neighborhood-related resources and walkability), the recorded postcode can be used in further studies.

Additionally, we offered only an abridged list of design components for an app, rather than all components that are conceivable in principle. The additional demonstration of mock-ups and prototypes to determine the preferences and desires of the participants might be useful in future surveys. Although user-centered methods for app design that combine different methods (eg, design thinking research) are time-consuming, they may improve the effectiveness of behavior-change support systems [44].

The description of the sociodemographic characteristics of our participants, grouped into smartphone users and non–smartphone users, showed that participants younger than the age of 70 years used smartphones much more often than older participants. Hence, the latter group of patients is hard to reach with mobile interventions. To improve the success of therapy for non–smartphone users, analog interventions (supporting medication use and the implementation of SET for older patients) should also be offered. Except for age, we found no noticeable differences between smartphone and non–smartphone users. The analysis of patient characteristics (ie, sex, education, burden, and health status) with respect to smartphone use, did not reveal any other significant differences between smartphone users and nonusers.

### Conclusion

This survey of patients with PAD indicates the necessity of improving the care situation of these patients. A need for support can be determined and identified with regard to educational and general support deficiencies. This need includes a better understanding of the prescribed medication and the necessary implementation of SET as a central pillar of the guideline-oriented care of patients with PAD.

There also exists a great interest in mobile support services. To improve the care situation of these patients, mobile interventions are promising. The large reach and wide availability of these interventions are major advantages.

## Appendix

Multimedia Appendix 1 Questionnaire.

Multimedia Appendix 2 Classification of topics.

## Abbreviations

mHealth: mobile Health
PAD: peripheral arterial disease
SEP: supervised exercise program
SET: supervised exercise therapy
