# Supplementary material for: Needs and Requirements in the Designing of Mobile Interventions for Patients With Peripheral Arterial Disease: Questionnaire Study
Source: JMIR Form Res. 2020 Aug 4;4(8):e15669. doi: 10.2196/15669 (PMC7435621; doi:10.2196/15669)
Supplement: Multimedia Appendix 1 [file formative_v4i8e15669_app1.pdf]

# Questionnaire to determine the need for support for patients with peripheral arterial disease (PAD)

We would like to expand our offers for patients with peripheral arterial disease (PAD). In order to design these offers close to your interests, you will find below some questions. With your participation in this survey you help us to gain findings on the supply of patients with PAD. All your answers will be kept strictly confidential.

We kindly ask you to fill in the questionnaire completely and to return it together with the signed declaration of consent at the registration desk of the Angiology Outpatient Clinic.

For better readability, only the male form will be used. The female form is always included.

**1. Please enter today's date.**

☐☐.☐☐.2018

---

**2. The results of this survey will also be evaluated for different age groups. Please state the year of your birth.**

Year of birth ☐☐☐☐

---

**3. Which gender do you have?**

Male . . . . . . . . . . . . . . . . . . . . . . . . . . . . . . . . . . . . . . . ☐
Female . . . . . . . . . . . . . . . . . . . . . . . . . . . . . . . . . . . . . ☐

---

**4. Please enter the postcode of your main residence.**

☐☐☐☐☐

# BURDEN AND DISEASE

**5. How strongly do you feel burdened by the disease PAD?**

| not at all | slightly | moderately | quite | very | No statement |
|---|---|---|---|---|---|
| ☐ | ☐ | ☐ | ☐ | ☐ | ☐ |

**6. How much are you burdened by the following diseases?**

| | not at all | slightly | moderately | quite | very | Not applicable |
|---|---|---|---|---|---|---|
| Diseases of the musculoskeletal system | ☐ | ☐ | ☐ | ☐ | ☐ | ☐ |
| Mental illnesses (e.g. depression etc.) or behavioural disorders | ☐ | ☐ | ☐ | ☐ | ☐ | ☐ |
| Diseases of the cardiovascular system | ☐ | ☐ | ☐ | ☐ | ☐ | ☐ |
| Hormonal nutritional or metabolic diseases | ☐ | ☐ | ☐ | ☐ | ☐ | ☐ |
| Diseases of the digestive system | ☐ | ☐ | ☐ | ☐ | ☐ | ☐ |
| Respiratory illnesses | ☐ | ☐ | ☐ | ☐ | ☐ | ☐ |
| Reproductive problems | ☐ | ☐ | ☐ | ☐ | ☐ | ☐ |
| Diseases of the central nervous system | ☐ | ☐ | ☐ | ☐ | ☐ | ☐ |
| Cancer | ☐ | ☐ | ☐ | ☐ | ☐ | ☐ |
| Kidney diseases | ☐ | ☐ | ☐ | ☐ | ☐ | ☐ |

**7. How much are you burdened by the following circumstances?**

| | not at all | slightly | moderately | quite | very | Not applicable |
|---|---|---|---|---|---|---|
| being constantly available for the family | ☐ | ☐ | ☐ | ☐ | ☐ | ☐ |
| family members in need of care or who are seriously ill | ☐ | ☐ | ☐ | ☐ | ☐ | ☐ |
| conflicts with other family members | ☐ | ☐ | ☐ | ☐ | ☐ | ☐ |
| balancing family and work | ☐ | ☐ | ☐ | ☐ | ☐ | ☐ |
| housekeeping | ☐ | ☐ | ☐ | ☐ | ☐ | ☐ |
| financial worries | ☐ | ☐ | ☐ | ☐ | ☐ | ☐ |
| low recognition of domestic and family work | ☐ | ☐ | ☐ | ☐ | ☐ | ☐ |
| sole responsibility of adolescent child(ren) | ☐ | ☐ | ☐ | ☐ | ☐ | ☐ |
| conflicts with partner or ex-partner | ☐ | ☐ | ☐ | ☐ | ☐ | ☐ |
| unwanted solitude and loneliness | ☐ | ☐ | ☐ | ☐ | ☐ | ☐ |
| parenting problems, conflicts with child(ren) | ☐ | ☐ | ☐ | ☐ | ☐ | ☐ |
| child(ren) that is/are chronically ill or disabled | ☐ | ☐ | ☐ | ☐ | ☐ | ☐ |

**8. Which aspect of your peripheral arterial disease (PAD) burdens you the most?**

____________________________________________

____________________________________________

# SUPPORT

**9. Do you currently take any medication that also serves to treat PAD?**

Yes . . . . . . . . . . . . . . . . . . . . . . . . . ☐→ How many different types of medication are you taking?

______________________________

No . . . . . . . . . . . . . . . . . . . . . . . . . ☐
I don´t know . . . . . . . . . . . . . . . . ☐

---

**10. Did your doctor explain to you why this medication is important?**

Yes . . . . . . . . . . . . . . . . . . . . . . . . . . . . . . . . . . . . . . . . . ☐
No . . . . . . . . . . . . . . . . . . . . . . . . . . . . . . . . . . . . . . . . . ☐
I don´t know . . . . . . . . . . . . . . . . . . . . . . . . . . . . . . . ☐

---

**11. What has helped you the most in dealing with your disease so far?**

________________________________________________________

________________________________________________________

---

**12. Would you like to have more support in dealing with your PAD?**

Yes . . . . . . . . . . . . . . . . . . . . . . . . . . . . . . . . . . . . . . . . . ☐
No . . . . . . . . . . . . . . . . . . . . . . . . . . . . . . . . . . . . . . . . . ☐
I don´t know . . . . . . . . . . . . . . . . . . . . . . . . . . . . . . . ☐

# WALKING-TRAINING (SET)

Guided walking-training - also referred to as supervised exercise therapy (SET) - is a cornerstone in the treatment of PAD and at least as effective as medication. In the following you will find some questions concerning the support of walking-training and possible forms of support for walking-training.

**13. Has supervised walking-training for the treatment of PAD ever been recommended to you?**

Yes . . . . . . . . . . . . . . . . . . . . . . . . . . . . . . . . . . . . . . . . ☐
No . . . . . . . . . . . . . . . . . . . . . . . . . . . . . . . . . . . . . . . . . ☐
I don´t know . . . . . . . . . . . . . . . . . . . . . . . . . . . . . . . ☐

**14. Do you have any idea what supervised walking-training for patients with PAD might be?**

Yes . . . . . . . . . . . . . . . . . . . . . . . . . . . . . . . . . . . . . . . . ☐
No . . . . . . . . . . . . . . . . . . . . . . . . . . . . . . . . . . . . . . . . . ☐
I don´t know . . . . . . . . . . . . . . . . . . . . . . . . . . . . . . . ☐

**15. How likely would you make use of the following offers?**

| | not at all | a little | moderately | quite likely | very likely |
|---|---|---|---|---|---|
| Training group with instructions | ☐ | ☐ | ☐ | ☐ | ☐ |
| Training via smartphone | ☐ | ☐ | ☐ | ☐ | ☐ |
| Online platform including exchange with other affected patients | ☐ | ☐ | ☐ | ☐ | ☐ |
| Home training exercises | ☐ | ☐ | ☐ | ☐ | ☐ |
| Support group | ☐ | ☐ | ☐ | ☐ | ☐ |
| Lectures and information events | ☐ | ☐ | ☐ | ☐ | ☐ |
| Information material | ☐ | ☐ | ☐ | ☐ | ☐ |

**16. How many meters can you walk currently without pain?**

- More than 1000 meters .......................... ☐
- More than 200 meters .......................... ☐
- Less than 200 meters .......................... ☐
- I don't know .......................... ☐

---

**17. What kind of walking-training would you be interested in?**
*(multiple answers are possible)*

- Group training .............. ☐
- Individual training .......... ☐
- Smartphone based training .. ☐ ↦ Which type would you prefer?
  in a group ........ ☐ alone ........ ☐

---

**18. Do you currently perform supervised walking-training?**

- Yes ........................ ☐ ↦ continue with question **20**
- No ........................ ☐ ↦ continue with question **19**
- No statement .............. ☐

**19. What are the reasons you are not practicing walking-training?**
*multiple answers are possible*

________________________________________

________________________________________

________________________________________

**20. On average, how much time per week could you spend on walking-training?**
*Choose the answer that in your option applies the best*

- More than 3 times 60 minutes ☐
- 3 times 60 minutes ☐
- 3 times 30 minutes ☐
- Less than 3 times 30 minutes ☐
- I don't know ☐

---

**21. Please indicate how satisfied you are with your medical care.**

| ☺ | 🙂 | 😐 | 🙁 | ☹ | Not sure |
|---|---|---|---|---|---|
| ☐ | ☐ | ☐ | ☐ | ☐ | ☐ |

## APP-USAGE

**22. Do you use a smartphone?**

- Yes ☐ ↦ continue with question **23**
- No ☐ ↦ continue with question **25**
- No statement ☐

**23. Have you ever heard of smartphone apps that aim to improve your health?**

Yes ☐ → continue with question **24**

No ☐ → continue with question **25**

No statement ☐

---

**24. Do you use such an app and if so, which one?**

Yes, namely: ____________________

No ☐

No statement ☐

**25. How <u>relevant</u> are the following categories regarding health apps to support patients with peripheral arterial disease?**

| | not at all | a little | moderately | quite | very | I don´t know |
|---|---|---|---|---|---|---|
| Get information about walking-training | ☐ | ☐ | ☐ | ☐ | ☐ | ☐ |
| Get feedback about walking-training | ☐ | ☐ | ☐ | ☐ | ☐ | ☐ |
| Receive suggestions for walking-training goals | ☐ | ☐ | ☐ | ☐ | ☐ | ☐ |
| Be able to choose walking-training goals | ☐ | ☐ | ☐ | ☐ | ☐ | ☐ |
| Be reminded of the execution of walking-training | ☐ | ☐ | ☐ | ☐ | ☐ | ☐ |
| Be able to interact with other people with PAD | ☐ | ☐ | ☐ | ☐ | ☐ | ☐ |
| Be able to interact with doctors or therapists | ☐ | ☐ | ☐ | ☐ | ☐ | ☐ |

**26. Would you be interested to participate in a study that deals with the support of walking-training for PAD patients??**

Yes . . . . . . . . . . . . . . . . . . . . . . . . . . . . . . . . . . . . . . . . ☐
No . . . . . . . . . . . . . . . . . . . . . . . . . . . . . . . . . . . . . . . . ☐

No statement . . . . . . . . . . . . . . . . . . . . . . . . . . . . . . ☐

---

**27. Are you interested in participating in a study, which examines the support of supervised walking-training for patients with PAD?**

Yes . . . . . . . . . . . . . . . . . . . . . . . . . . . . . . . . . . . . . . . . ☐
No . . . . . . . . . . . . . . . . . . . . . . . . . . . . . . . . . . . . . . . . ☐

No statement . . . . . . . . . . . . . . . . . . . . . . . . . . . . . . ☐

## GENERAL INFORMATION

**28. What is your highest school leaving qualification?**

- Left without any qualifications ☐
- Lower secondary or primary school qualification ☐
- Polytechnic Advanced Level (8th/9th grade) ☐
- Secondary school/ polytechnic secondary school (10th grade) ☐
- Higher secondary school (11th / 12th grade) ☐
- General university entrance qualification (12th / 13th grade) ☐

- No statement ☐

---

**29. Have you completed a vocational training?**

- Yes ☐
- No ☐

- No statement ☐

---

**30. Do you have a university degree?**

- Yes ☐
- No ☐

- No statement ☐

---

**31. Are you currently employed?**

- Yes, I work full time (36 and more hours per week) ☐
- Yes, I work part time (less than 36 hours per week) ☐
- I am a housewife/ househusband ☐
- I am currently jobseeking/ unemployed ☐
- No, I am pensioned due to my illness(es) ☐
- No, I am pensioned due to my age ☐

Other: ______________________

# Thank you very much for your participation!
