# Supplementary material for: Needs and Requirements in the Designing of Mobile Interventions for Patients With Peripheral Arterial Disease: Questionnaire Study
Source: JMIR Form Res. 2020 Aug 4;4(8):e15669. doi: 10.2196/15669 (PMC7435621; doi:10.2196/15669)
Supplement: Multimedia Appendix 2 [file formative_v4i8e15669_app2.docx]

**Appendix 2.** Thematic structure of the questionnaire

*Need for support*

To determine if there is a general need for support, the following question was used: “Would you like to have more support in terms of dealing with your PAD?” Other questions that were asked regarding support or need for action focusing on the quality of patient care were as follows:

- “Do you currently take any medication that also serves to treat PAD?”
- “Did your doctor explain to you why this medication is important?”
- “Has supervised walking training for the treatment of PAD ever been recommended to you?”
- “Do you know what supervised walking training might be?”
- “Do you currently perform supervised walking training?”

These questions addressed the level of information, knowledge and specific actions that might be relevant for patient-centred care. To answer the questions, the study participants were offered the answer options “yes”, “no” and “do not know” or “no information”.

*Satisfaction with healthcare situation*

Satisfaction with health care in general (Q21 “Please indicate how satisfied you are with your medical care”) was surveyed on a 5-level scale from 1=very satisfied to 5=very dissatisfied. The answer scale was illustrated with five "smileys", whose facial expressions were adapted accordingly. In addition, the answer option "not sure" was offered.

*Sociodemographic characteristics*

Participants reported their sex, year of birth, level of education, employment status and place of residence (postal code). The International Standard Classification of Education (ISCED) was used to determinate educational attainment ^1^, which was classified by the attended primary, secondary, tertiary, and other kinds of qualified education such as vocational trainings. We assigned these levels to equivalent durations of school years ^2,3^. Up to 9 years of school attendance were equal to an ungraduated or lower level of secondary education, 10 – 11 years were equal to a lower secondary level of education, 12 – 13 years were equal to an upper secondary level of education or vocational training, 14 – 17 years were equal to an upper secondary level of education and additional vocational training, and more than 17 years were equal to an academic degree and/or additional vocational training ^1^.

*Burden of environmental conditions*

To determine how much the study participants were burdened by daily life, the respondents were asked to answer the question "How much are you burdened by the following circumstances?" with regard to different items: being constantly available for their family, family members who are in need of care or seriously ill, conflicts with other family members, balancing family and work, housekeeping, financial worries, low recognition of domestic and family work, the sole responsibility for adolescent child(ren), conflicts with their partner or ex-partner, unwanted solitude and loneliness, parenting problems/conflicts with their children, or child(ren) that is/are chronically ill or disabled. To answer this question, the participants were provided with five-point scales that covered an answer spectrum from 1=not at all burdened to 5=very burdened. Additionally, “not applicable” could also be selected.

*Burden of PAD and other diseases*

The burden of PAD (Q5 “How strongly do you feel burdened by PAD?”) was surveyed on a 5-point scale from 1=not at all to 5=very much. In addition, the answer option "no statement" could be selected. Additionally, participants were asked to answer, "How much are you burdened by the following diseases?” with regard to different types of diseases (see Appendix 1).

To answer these questions, the respondents were provided with five-point scales that covered an answer spectrum from 1=not at all to 5=very burdened, and “not applicable” was included as an answer choice.

*Pain-free walking distance*

To further characterize patients with PAD and to roughly estimate the severity of the disease, study participants made an additional self-assessment of their pain-free walking distance.

Therefore, Q16 categorized the pain-free walking distance based on the Fontaine classification ^4^: more than 1000 metres, more than 200 metres (but less than 1000 metres), and less than 200 metres.

*Clinical characteristics*

Clinical characteristics included the cardiovascular risk factors recorded by a physician, including arterial hypertension, hypercholesterolemia, smoking and overweight. PAD-related morbidities such as Fontaine stage, previous amputations, previous peripheral intervention or peripheral bypass grafting were investigated.

The severity of PAD was classified by a treating physician using the Fontaine stages ^4^: Stage I (corresponding to mild PAD), Stage IIa, Stage IIb, Stage III and Stage IV (corresponding to very severe PAD). ^4^

Additionally, pre-existing conditions such as atrial flutter, coronary artery disease, diabetes, chronic obstructive pulmonary disease, chronic kidney failure, and cerebrovascular disease were included in the analysis.

As part of standard clinical care, risk factors for PAD (such as smoking) were also assessed. Based on the participants’ reported height and weight, body mass index (BMI) was calculated. Individuals with a BMI between 25 and 30 kg/m^2^ were classified as overweight; those with a BMI >=30.0 kg/m^2^ were classified as obese.^5^

*Preferences in offers to support patients with PAD*

We asked patients about seven potential offers to support the implementation of walking training: (1) A training group with instructions, (2) training via a smartphone-app, (3) an online platform including an ability to interact with other affected patients (e.g., a forum), (4) an online platform with home training exercises, (5) a support group, (6) lectures and patient information events and (7) informational material. We measured the probability of making use of the proposed offers using five-point Likert scale items (e.g., “How likely is it that you would take advantage of the following offers?” with response options from “not at all” to “very likely”).

*Smartphone usage, knowledge about health apps and health app usage*

Smartphone usage (“Do you use a smartphone?”) was surveyed on a dichotomous “yes” or “no” scale. In addition, the answer option "no statement" could be selected.

The patients' level of knowledge about and use of health apps was also assessed based on the following questions: “Have you ever heard of smartphone apps that help you improve your health?” (Q23) and “Do you use such an app?” (Q24).

*Design categories in health apps to support patients with PAD*

We measured the relevance of potential design categories using five-point Likert scale items (e.g., “How important are the following categories for a health app to support patients with peripheral arterial disease,” with response options from “not at all” to “very relevant”).

1 International Standard Classification of Education (ISCED) 2011: UNESCO Institute for Statistics; 2012.

2 Hennig F, Fuks K, Moebus S, Weinmayr G, Memmesheimer M, Jakobs H, et al. Association between source-specific particulate matter air pollution and hs-CRP: local traffic and industrial emissions. Environ Health Perspect. 2014;122:703–10. doi:10.1289/ehp.1307081.

3 Kälsch H, Hennig F, Moebus S, Möhlenkamp S, Dragano N, Jakobs H, et al. Are air pollution and traffic noise independently associated with atherosclerosis: the Heinz Nixdorf Recall Study. Eur Heart J. 2014;35:853–60. doi:10.1093/eurheartj/eht426.

4 Aboyans V, Ricco J-B, Bartelink M-LEL, Björck M, Brodmann M, Cohnert T, et al. 2017 ESC Guidelines on the Diagnosis and Treatment of Peripheral Arterial Diseases, in collaboration with the European Society for Vascular Surgery (ESVS): Document covering atherosclerotic disease of extracranial carotid and vertebral, mesenteric, renal, upper and lower extremity arteriesEndorsed by: the European Stroke Organization (ESO)The Task Force for the Diagnosis and Treatment of Peripheral Arterial Diseases of the European Society of Cardiology (ESC) and of the European Society for Vascular Surgery (ESVS). Eur Heart J. 2018;39:763–816. doi:10.1093/eurheartj/ehx095.

5 Kaphingst KA, Bennett GG, Sorensen G, Kaphingst KM, O'Neil AE, McInnis K. Body mass index, physical activity, and dietary behaviors among members of an urban community fitness center: a questionnaire survey. BMC Public Health. 2007;7:181. doi:10.1186/1471-2458-7-181.
